# A discussion of gender, ethnicity, and intersectionality, at the Serb Business Association forum

**DOI:** 10.3389/fsoc.2023.1231050

**Published:** 2024-01-04

**Authors:** Aleksandra Paravina

**Affiliations:** Department of Sociology, University of Helsinki, Helsinki, Finland

**Keywords:** Serb women, ethnicity, conversation analysis, gender, Croatia

## Abstract

Qualitative transdisciplinary research has contributed to the development of a dynamic scientific area that is best suited to analyze real-life data from real people. To determine how ethnicity and gender intersect to shape the social worlds of the participants in a Serb business discussion forum, I apply two theoretical orientations based on ethnomethodology: Conversation Analysis (CA) and membership categorization analysis (MCA). I analyze 15 videos with predominantly female Serb discussants. My findings from this project reveal a significant presence of stigma in the perceptions of Serb ethnicity in Croatia. I argue that due to patriarchal values as a type of cultural cache, Serb women experience demeaning gender categories in various areas of their lives. To improve this situation, it is suggested that there is a need for feminist ethics of care and a coalition with men.

## 1 Introduction

In this article, I analyze the intersectionality of gender and ethnicity through a biweekly Serb Business Association public discussion forum titled “Say What You Think!” (*Reci što misliš!*). The Serb Business Association, Privrednik (*srpsko privredno društvo*), is one of the oldest Serb philanthropic organizations that helps underprivileged but talented young people in Croatia. Established in 1897 by a businessman and philanthropist Vladimir Matijević (Radović, 2018), it was banned in 1940 but re-established in 1993. Privrednik has achieved remarkable and significant results during its existence; guided by the basic principles of work, savings, and honesty, and it managed to educate numerous generations of young people, providing them with support and prepared them for life’s challenges (Radović, 2018). As the name Business Association can be misleading, Olivera Radović, vice-president and the author, points out that, as with many other Privrednik activities, such as information or cultural programs, forums seek to educate scholarship holders and raise issues that aim to preserve their cultural and national identity. The situation of Serbs in Croatia is highly challenging, as they are often discriminated against and stigmatized in public. In response, Privrednik attempts to contribute to pluralization through forum discussions and media images about the Serbian community (Radović, 2022a).^1^

This analysis of Privrednik forum discussions emphasizes the importance of foregrounding gender and ethnicity and draws on the feminist theory of intersectionality that was developed by Black feminist scholars in the late 1980s and 1990s. These scholars were critical of “the tendency to treat race and gender as mutually exclusive categories of experience and analysis” (Crenshaw, 1989, p. 139). “Moreover, ignoring differences *within* groups contributes to tension *among* groups” which was problematic for identity politics where feminists sought to politicize the experience of women on an exclusive terrain from antiracists (Crenshaw, 1991, p. 1242). Some parallels can be found in the examples that Crenshaw (1991) offers in her analysis of Black women’s marginalization and in her position that racial and sexual subordination reinforce one another. One similarity to Crenshaw’s examples is the daily masculine violence that occurs in the Western Balkans, which is the “result of unemployment, poverty, and a lack of any social safety nets” (Dumančić and Krolo, 2017, p. 165). Violence also alters the social experience of ethnicity and the study by Golubović (2020, p. 546) on how it feels to be a Serb woman in Sarajevo Bosnia shows the cumulative sense of anxiety rooted in the experience of war. The narrative on ethnicity should therefore be viewed not only from the perspective of Serb men, just as Crenshaw (1991, p. 1299) calls for the reconceptualization of race as a coalition between men and women of color.

According to the 1946 Yugoslav Constitution (Bonfiglioli, 2020, p. 33), women as “equal citizens and workers” were provided with employment, education, independent income, and relief through socialized and collectivist reproductive work. Even though patriarchy was dismantled at the institutional level during the socialist period, it was not eradicated in all segments of society (Kupsjak, 2022, p. 209, see also Hammel, 1984; Pavićević, 2007). As part of her study on “Women and Industry in the Balkans” (2020, p. 37), Bonfiglioli notes that at the beginning, “only very poor village girls accepted to work in the factory” because the majority of local families rejected the possibility of young women working outside their homes. The rural population even considered bicycles as a means of transportation to be immoral and many women who worked both in the factory also worked in the fields (Bonfiglioli, 2020, pp. 38–39). As a result of the Croatian War of Independence (1991–1995), many references to Serb culture and socialism were officially prohibited (Paravina, 2022, see also Jovic, 2017). My research focuses on the experiences of women in the post-war context, a time when the Serb women in rural areas are struggling to secure sustainable employment.^2^ As a result of patriarchal values as a type of “cultural cache” (see also Dumančić and Krolo, 2017, p. 162), Serb women experience the “culture’s gender categories” (Stokoe, 2012b) as demeaning in various aspects of their lives, which particularly pertains to demeaning care work such as the provision of food by women. Women engage in food preparation at home and also contribute to food production through their farm work and other agricultural jobs. Since the 1990s, perspectives on formal and informal care research have been more concerned with intersectionality and have encompassed gender, ethnicity, age and class (Anttonen and Zechner, 2011, p. 32). To improve this situation, additional coalitions with men need to be formed as well as adopting the feminist ethics of care.

The objective of this analysis is to investigate (a) how Serb women orient to both gender and ethnicity and (b) how ethnicity and gender intersect to shape the social worlds of the participants in the public discussion forums of the Serb Business Association. In other words, as Schegloff (1991, p. 51) argues, to orient is “to show how the parties are embodying for one another the relevancies of the interaction and are thereby producing the social structure.” To analyze the data in “talk in interaction,” we must demonstrate that the parties were, with each other, “demonstrably oriented to those aspects of who they are” and those aspects of their context that are “respectively implicated in “social structures”” (Schegloff, 1991, p. 52). The concept of “social structures” refers to power and status as well as to their “distribution among social formations such as classes, ethnic groups, age grade groups, gender, and professional relations” (Schegloff, 1991, p. 45). I adopt the principles of ethnomethodology (EM) as well as Garfinkel (1986) work known as the “studies of work” program which attempts “to discover members’ methods of accomplishing the complex tasks of everyday life, including methods of reasoning, methods of interpretation” (Psathas, 2006, p. 255). The objective is to discover the patterned or sequential character of these practices or their “orderliness.” In this study, I extend my analysis by using the EM/CA proposed by Arminen (2004, p. 685) “On the weakness of institutional rules” where a model of accountable action is presented through the “reflexive relationship among rules, their application and the setting.” As the Serb Business Association is an institution, it would be expected that it has specific rules of conduct. As Garfinkel (1986, p. 2) remarks in order to “make judgments about institutions, or other social phenomena which display ‘regularities,’ the criteria and concepts in use must be understood ‘in relation to the rules governing sociological investigation’.” Since this forum does not have any formal rules, I have utilized minority rights as my criteria for sociological investigation. I identify the moments of discussion when parties discuss minority rights, and I focus on the manner in which they formulate and reformulate these rights.

Due to the circumstances of the last war and the pressures on the Serb minority to assimilate in Croatia, these rights are not always respected and available (Paravina, 2022). The guests who most frequently attended the forum were Croat female intellectuals. This could also be attributed to the nature of the public discussions, which criticize Croatian society, particularly the issues related to abortion rights, equality, and non-discrimination. The topics discussed in the forum are related to the Charter of Fundamental Rights of the European Union (EU) which protects minorities as well as to the Croatian Constitution which guarantees those rights for the Serb minority. I have therefore specifically selected several discussions with Serb women who either live in rural areas or who, although they are educated, have roots in the country. In addition, I have adopted an ethnomethodological approach that was developed in the early 1960s, MCA, combining this theoretically with the orientation by Sacks (1992) and *CA.* While “sequential analysis” has been the primary focus of CA, the membership categorization practice addresses “contextual factors” (Psathas, 1999, p. 141). My data feature examples where “MCA serves to reveal the ways in which populations and constituent identity groups are categorized, morally constituted and accounted for in practice” (Housley and Fitzgerald, 2009). In that sense, this study seeks to build on some uneasy discussion related to combining CA with MCA, particularly mentioning Heritage (2012, p. 80) and his viewpoint that the “next turn is the ‘go to’ place for the empirical warrant for a claim” in relation to requests for information such as assertions as questions or negative interrogatives.

The requirement for the ethnomethodological approach is that any pre-formulations related to aspects such as identity or personal characteristics must be set aside and “the relevance of such matters be shown demonstrably” (Psathas, 1999, p. 141). Other scholars, such as Stokoe and Attenborough (2015, p. 4), argue that in MCA, “sequential matters come first.” This was demonstrated in studies by Stokoe (2012a,b) that investigated institutional settings, larger corpuses with sequential organization, and environments in which categories appeared in “remarkably similar ways, across similar action-oriented environments.” Thus, the criticism that by combining CA and MCA, “categorial phenomena are ‘disorderly’” has been refuted (Stokoe and Attenborough, 2015, p. 3). The ethnomethodology approach examines the “rational properties of indexical expressions,” as Garfinkel (1967, p. 11) emphasizes, and as categories are inference rich, we can also say that the “links between categories, actions, and predicates” cannot be decontextualised (Stokoe and Attenborough, 2015, p. 6). Stephen Hester studied “categorial formulations of deviance” in educational settings and discussed “the problem of ‘culturalism’” and the “occasionality of categorical formulations” in his unpublished manuscript (Francis and Hester, 2017, p. 56). Nevertheless, Francis and Hester (2017) reviewed Hester’s unpublished empirical work and concluded that “category-words do not have stable, uni-referential meanings”; as such, they cannot be decontextualized completely.

The next section presents a description of the design and methodology of this study. I first explain the data collection that consists of recordings between 2014 and 2022 from Privrednik’s public discussions. I adopt the analytical method of EM/CA in combination with MCA to analyze and transcribe recordings. The intersection of gender and ethnicity reveals a rather tentative coalition between Serb men and women, rather demeaning gender categories in a culture as well as stigma. The analysis will be summarized and discussed through four examples that reveal how participants perceive ethnic identity differently.

## 2 Materials and methods

My empirical data originate from the YouTube videos of the biweekly live public discussion forum in Privrednik, Zagreb, Croatia.^3^ Through this forum, participants are encouraged to discuss a variety of topics related to the development of society, ranging from issues of human rights and minority rights, to discrimination and historical revisionism. Several topics are addressed, one of them being Croatia’s anti-fascist legacy. As this anti-fascism is virtually inseparable from its communist legacy, most of the disagreements between the political Left and the Right are related to renaming the streets connected to the Communist Party or Partisan military units where Serbs fought in great numbers during WWII (Šakaja and Stanić, 2011; Vretenar and Krajina, 2016). Furthermore, some Croatian nationalists deny the genocide of the Serbs in the Independent State of Croatia (ISC) that existed from 1941 to 1945 (McCormick, 2008; Kasapović, 2018; Goldstein, 2023). The ISC consisted of the modern-day states of Croatia, Bosnia and Herzegovina as well as the Serbian province of Srem. The “fascist Ustasha regime implemented policies of racist extermination against another Slavic people: the Serbs” (Connelly, 1999, p. 5; Bartulin, 2013; Yeomans, 2013; Mrkalj, 2023). The memory of WWII is reinterpreted and “instrumentalized for narrow nationalist agendas,” so that in Croatia, the crimes committed by the Ustaša regime are relativized to the extent that even the suffering at the Jasenovac concentration camp is downplayed in one textbook by describing it “as a place where people died from poor hygiene and infectious diseases,” while in all textbooks, Croats are included in the list of victims, “attempting to evade responsibility for the committed crimes” (Trošt and David, 2022, p. 12, p. 5). Serbia, however, uses Jasenovac to “counterbalance Srebrenica and insist on Serb victimhood” (Trošt and David, 2022, p. 6).^4^Although there is ample evidence that Ustaše did “carry out a mass murder of Serbs against the German will,” (Korb, 2010, p. 146) it is nonetheless highly disconcerting to relieve Ustaše from any responsibility for the genocide as there was sufficient evidence for that as far back as in 1941 (Goldstein, 2007; McCormick, 2008). We must also remember that this genocide had a far wider impact on society, including the displacement of many people and the destruction of many families (Livada, 2018).

The video recording of the first Privrednik public discussion forum titled “Say What You Think!” (*Reci što misliš!*) began in 2013, and I have selected 15 out of the 112 recordings that were aired between 2013 and 2022. The duration of each of these recordings is approximately 1 h. In addition, I have also included one press conference discussion of the results of Privrednik’s recent media project titled “Let us Get to Know (One Another) in Order to Understand Each Other” (Radović, 2022b). When selecting the recordings, I intentionally chose featured (1) Serb women from a variety of backgrounds and ages and (2) discussions concerning ethnicity such as gender equality, minority rights, education, and work. The online automated transcription service Sonix^5^ was used, and I transcribed 6 h, 12 min, and 4 s of the material relevant to my study. The Sonix was only used for the transcription of the verbal material that was not in the actual CA transcript that I had to transcribe manually. The advantage was that as YouTube videos can be automatically accessed through the shortened weblink, it was easier to review sections of the literated verbal material with a highlighting tool that additionally indicates precise timecodes while simultaneously playing the video. In addition, the Sonix software can color code individual speakers and display timecodes when they begin to speak. It is also possible to “strike through crossed out selected text” in the transcription (see Footnote 5). However, this is not reversable, which means that time codes will change, and the corresponding video section will be cut as well within the Sonix interface.

There was also a possibility to translate the literation, but the only language available for that was Croatian, not Serbian. This meant that I needed to edit some of the transcriptions according to the manner that some discussants spoke. Although they were all Ijekavian speakers, their use of vocabulary, phrasing, or idiomatic expressions varied, which meant that the transcription confidence levels sometimes reached 80 percent while they were lower at other times. For example, the word “*čovjek*” (Cro. or Srb.) to refer to a person or a man is used extensively in idiomatic expressions or phrases such as “(*kažem ti*) *kao čovjeku*” meaning “to say it honorably, sincerely, without preconceived notions” [Hrvatski jezični portal (HJP) 2023]. In a dictionary of the Croatian language published in 1998 (Anić, 1998), none of the four meanings of the word “*čovjek*” contains a note on being limited to the male gender (Kordić, 2001, p. 472). Even though the dictionary states that only those referring to psychological qualities apply to both sexes (Kordić, 2001, p. 473), this is not the case in Serbian, as the word has retained the association to the adult male gender as well as to “people in general.” What is disappearing from contemporary usage in Croatian [and Serbian] is the referential “*čovjek*” with the meaning “husband” (Kordić, 2001, p. 472). The quality of audio files also varied.^6^ The videos feature a variety of guests, the majority of whom are from the Serb minority, but there are also some Croats among them. Although the speakers are from a variety of backgrounds, most are highly educated in that the topics of discussion require them to have a high level of knowledge from their professions. This qualitative transdisciplinary research provides the “possibility to interpret research data gathered in real-life contexts,” thus creating a dynamic knowledge exchange between scientists and, [in this case, stigmatized minority] with “real people and their life experiences” in a heterarchical manner, understanding that these findings are common goods” (Filipović, 2019, pp. 494, 496). The recordings are publicly accessible on Privrednik’s YouTube channel (Privrednik 2023).

The theory for this analysis combines EM/CA with membership categorization analysis (MCA). As it is difficult to predict when someone will begin talking about gender and ethnicity, it appeared to be relevant to my study to use both categorization and sequential analyses. We can define CA as “a program of “reverse engineering” which analyzes interactional practices in order to articulate and respecify the generic building blocks of social interaction” (Arminen, 2016, p. 5). Some characteristics of CA are that it can be applied in an analysis of institutional settings, it does require background knowledge because “in institutionally distinct settings, the recognizability of parties’ actions may not be pre-given, as the details of actions may be informed by expert knowledge or organizational specifics not known to outsiders” (Arminen, 2016, p. 16). Another important aspect of CA is that it “treats talk and social interaction as a sufficient object for analysis, rather than as a window to wider social processes or as a medium for data collection” (Arminen, 1999, p. 251). In the context of this study, I have taken into consideration the details in interaction, turn-taking, and in the overall *structures* of social action (Atkinson and Heritage, 1984; Wooffitt, 2005). At the same time, I have followed membership categorization analysis (MCA) which has had strong focus on categorical concerns rather than on sequential practices that are common in CA (Stokoe, 2012b, p. 236). The important distinction for MCA is how gendered “common sense” is constructed, challenged and maintained; how gender is realized and made morally accountable (Stokoe, 2012b, p. 235).

The patterns that emerged in the discussion data resemble the ways rules are rephrased in the peer group interaction in Arminen (2004) study of the rules for group therapy: (1)“Contextualization of rules” (2) “Downgrading the rules through reformulations” (3) “Reading the rules plainly” and (4) “The tension between two rule authorities on the surface: laughter.” Here the ethnomethodological focus has been on the model of accountable action rather than on the rule-following model (Arminen, 2004, p. 684). As the purpose of Privrednik’s forum discussions is to preserve Serb cultural and national or ethnic identity, my analysis has taken into account minority rights as a pluralistic and an abstract social order (Kim, 2003, p. 20). Privrednik adheres to this in the structure of the interaction and the content of the public forum. Humankind relies on the basic humanity of others rather than their superficial roles, but the notion of humanity is highly abstract and vague (Kim, 2003, p. 27). For Goffman, “the self is not a fortress, but rather a small open city,” which means that whenever the city is threatened, the self is reborn and every new situation involves a constant process of redefining the self (Kim, 2003, p. 76). Through Goffman (1963) study of stigma, I elaborate on the reasons that the Serb minority members are disqualified from full social acceptance or on why their social identity as Serbs is perceived as less desirable, even dangerous, bad, or weak. This will be discussed in more detail in the discussion section of this article.

CA begins with a minute transcription of the data, following the guidelines originally established by Jefferson (2004). An overview of the transcription symbols is provided in Supplementary material. The discussions are in Croatian/Serbian and the analysis can be followed with the translation. The data analysis is based on the original languages of Croatian/Serbian as well as the idiomatic/free translation in English (Arminen, 2016, p. 66). The primary focus of interaction is speech, but I have also included visual glosses such as gazes, gestures, and postures.

## 3 Results

When conducting an empirical study of an institutional setting, practices can be clearly identified by focusing on interactional patterns and generic phenomena (Arminen, 2016, 83). For the current analysis, I have selected the four most clearly representative cases. These include (1) a plain reading the rules and in this case, minority rights; (2) personalized presentations of ethnicity and a contextualization of the Serb ethnicity; (3) a downgrading of the ethnicity through reformulation; and (4) “The discussion is closed with cases in which the participants’ ambivalence toward rules (in this case, minority identity and gender equality) surfaces through laughter” (Arminen, 2016, 78). Most of the discussions (9 out of 15) are moderated by a journalist, Saša Kosanović, who prefers a slightly humorous and sometimes confrontational style that suits the aims of discussing how “people justify their beliefs, deliberate about difficult matters, or give accounts of their opinions” (Brinkmann, 2013, 60). In the following sections the analysis will be summarized and discussed through four examples.

### 3.1 Reading the ethnicity and gender rights plainly

Although expressing one’s ethnicity freely might appear to be a general moral norm, or in this case, rights read plainly, Arminen (2004, p. 693) argues that although it may be difficult to find substantial grounds for claims about the instructor’s interpretation of the rule, one means of determining the interpretation by the instructor is to observe whether the instructor orients to different types of rules differently. In this instance, the moderator in Excerpt 1 “Screening of the film “S*rbenka*” alludes to sexual identity rights.^7^

**Figure 1:**
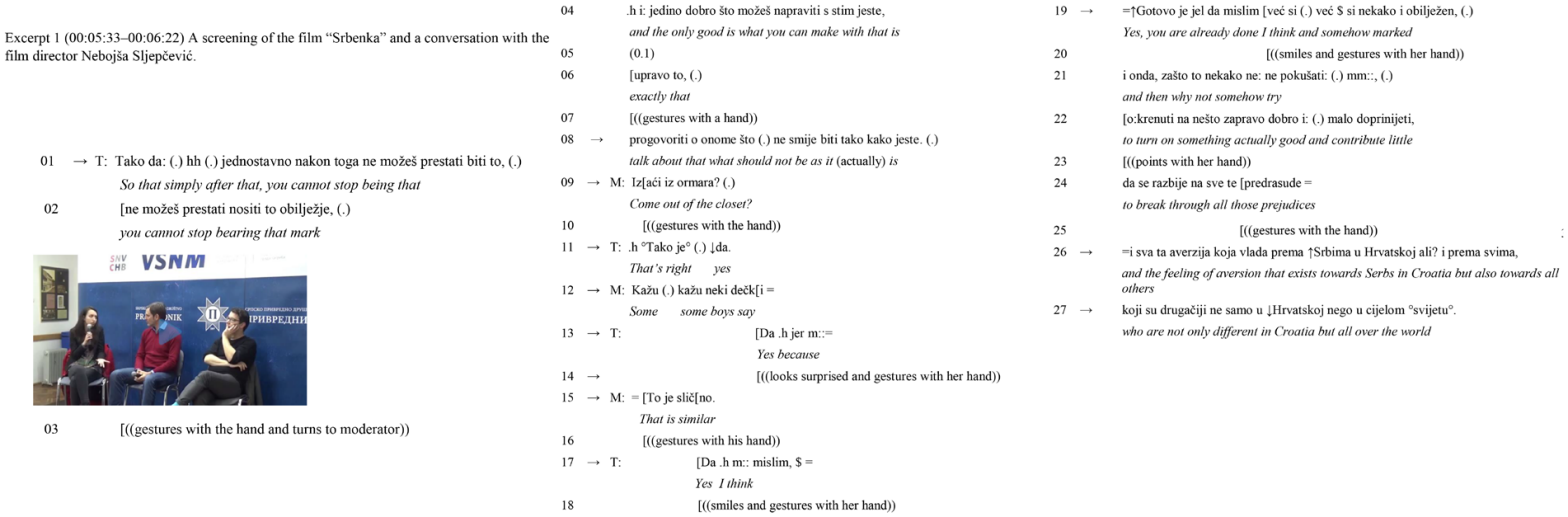
Excerpt 1 – Video participants include film director Nebojša Sljepčević, actress Tatjana Dragičević, and moderator Saša Kosanović (Privrednik, 2018c).

The discussants are: actress Tatjana Dragičević, and moderator Saša Kosanović (Privrednik, 2018c; Figure 1).

In line (1.1), Tatjana states, “So that simply after that, you cannot stop being that.” Tatjana asserts that Serb ethnicity forms part of a tacitly assumed cultural competence that guides and evaluates any social behavior and while it is not always followed, it is part of the general ethical guidelines in everyday life (Arminen, 2004, p. 694). In response to her statement in line (1.8): “Talk about that what should not be so, as it (actually) is,” the moderator formulates a categorial upshot in line (1.9), “Come out of the closet?” In this manner, the moderator proposes that ethnic and sexual minorities typically behave similarly in that they are more careful in expressing their identity. The “common knowledge component” in this categorial practice (Stokoe, 2012b, p. 240) is Tatjana’s response in line (1.11) “That’s right, yes.” Within the next turn, we also see that the moderator “builds a gendered context out of a previously non-gendered one, replacing the interviewer’s gender-neutral category” from line (1.9) “Come out of the closet” to line (1.12) “Some boys say” (Stokoe, 2012b, 239). In the next turn, it is evident that Tatjana is at first somewhat surprised by the moderator’s comparison to gay men. Tatjana subsequently attempts to respond by slightly mumbling in line (1.13), “yes, because,” before being interrupted by the moderator, who states in line (1.15), “that is similar.” However, during a subsequent turn, Tatjana takes the option of expanding on her feelings of discomfort (Wooffitt, 2005, p. 173), and states in a smiley tone of voice line (1.19), “Yes, you are already done I think and somehow marked” and further she asserts that in that sense, why not try to do something good and break through line (1.26) “the feeling of aversion that exists towards Serbs in Croatia, but also towards all others.” By stating this, she also expands on the comparison between being ethnic and being LGB to all line (1.27) “who are not only different in Croatia but all over the world.”

According to Article 21 of the EU Charter of Fundamental Rights, discrimination based on ethnicity and sexual orientation is prohibited (European Union, 2016). The moderator explicates that particular right by stating that being open about one’s ethnic identity is like “coming out of the closet.” In this manner, the moderator points out a particular institutional contingency that does not prevail outside (Arminen, 2004, p. 694); in other words, people will carefully consider where they feel safe to openly express their sexual orientation, they are likewise more reserved about their ethnic identity as well. Nevertheless, the comparison of an ethnic woman to a gay man in this context is rather problematic and will be discussed further.

### 3.2 Personalized presentations and contextualization of the Serb ethnicity

This video excerpt is from a press conference for the media project titled “Let us Get to Know (One Another) in Order to Understand Each Other.” Bogdanka Srdić Vulpe, who came to Zagreb originally from the region of Lika in Croatia in 1964, is explaining what it was like for Serbs when they were summoned to do factory work in the bottling plant in the 1990s during wartime.

**Figure 2:**
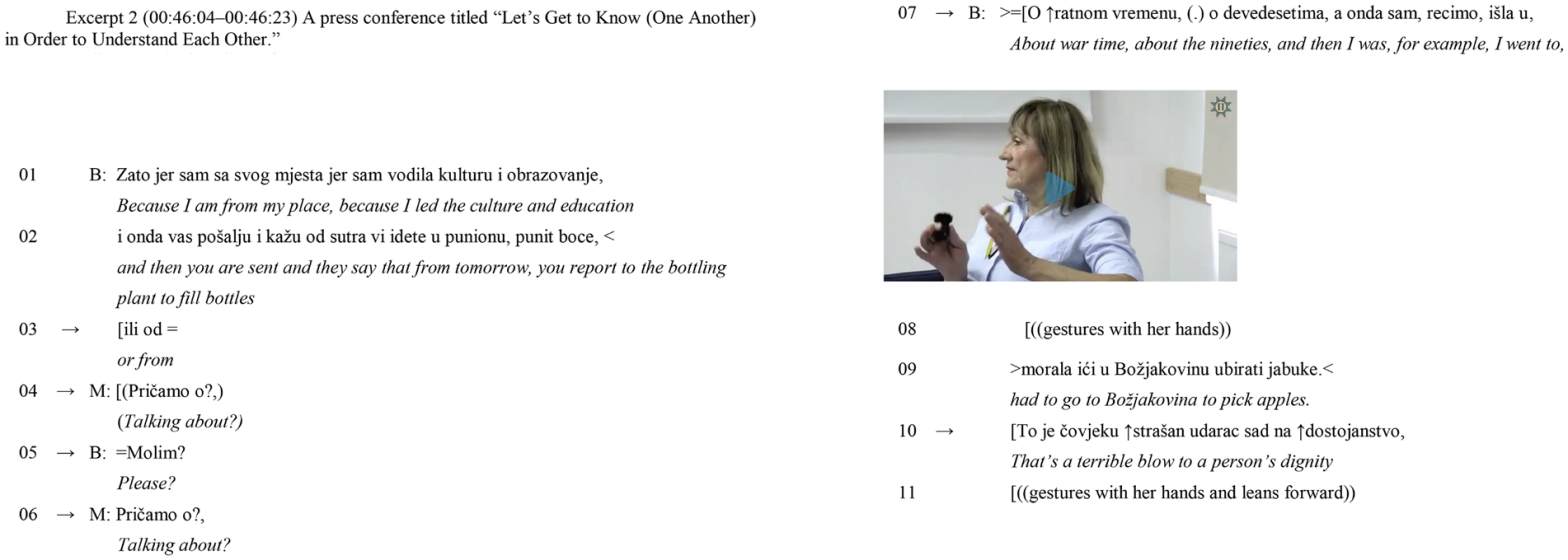
Excerpt 2 – Video participants include project manager Nikola Lunić, editor of P-portal, Olivera Radović, administrator Maša Samardžija, Privrednik’s female activists and podcast guests Slavica Beljak and Bogdanka Srdić Vulpe (Privrednik, 2022).

The discussants are Bogdanka Srdić Vulpe, Privrednik’s female activist and Maša Samardžija, an administrator (Privrednik, 2022; Figure 2).

When we analyze the turn-taking in this excerpt line (2.2), Bogdanka states, and then you are sent and they say that from tomorrow, you report to the bottling plant to fill bottles, she is “inserting background information,” and orients to the younger Serbs or Croats who have not experienced war as non-knowing individuals (Arminen, 2004, p. 688). One of the young minority representatives, Maša, who is following Bogdanka’s statements, inquires in line (2.4), “Talking about?” while speaking in overlap with Bogdanka’s production of the word “Or,” which makes it unclear. We can see that line (2.4) by Maša: “Talking about?” is designed to make Bogdanaka uncomfortable because structurally, it comes after Bogdanka has initiated her turn that is legitimately hers to take (Wooffitt, 2005, p. 174). It is clear that Maša does not align with the trajectory of Bogdanka’s statements.

Bogdanka attempts to contextualize the experience of being a Croatian Serb in the 1990s. In lines (2.7–2.9), she states: “About the war time, about the nineties, and then I was, for example, I went to, had to go to Božjakovina to pick apples.” As an educated woman, who was also working in culture, Bogdanka’s social standing was shattered during the war because she was forced to work in a bottling plant and to pick apples. In line (2.10), she observes: “That’s a terrible blow to a person’s dignity.” The feeling of stigmatization was exacerbated by the fact that all of the others who worked with her were either Serbs or their spouses and that reminded her terribly of the Jasenovac concentration camp. We could say that Bogdanka is alluding to Article 5 of the EU Charter of Fundamental Rights which states that: “No one shall be required to perform forced or compulsory labour.” In the study of “contextualizing the rules,” in order to make institutional rules understandable to newcomers, Arminen (2004, p. 689) identifies three techniques that involve the descriptive detailing of a rule. The first technique is to provide background information. The second is to explain the meaning of the rule. The third technique is to propose when, where, and how to use it. Although this is a slightly different context, we can see how in Excerpt 2, Bogdanka provides background information and the meaning of “Prohibition of slavery and forced labour” as a means to fight discrimination towards Serbs.

### 3.3 Downgrading of ethnicity rights through reformulation

This excerpt titled “If they remember me on Women’s Day, they do” is a public discussion on International Women’s Day that was important during the socialist era but has not retained that significance today. Sonja Leka, who is participating through Skype, talks about the difficult situation of Serb women in rural Lika near the well-known Plitvice Lake nature park, where she founded Citizens of Tara, a handicraft association. However, the management of the National Park has opposed attempts by the Association to establish a sales point near Plitvice Lakes. According to Sonja lines (3.2–3.4), “We deliberately did not put anything national in the name because we wanted to be citizens, because we are citizens of all of Croatia.” With this statement, she nods slightly as if to accentuate or signal her words. This gives the impression that there is a reference to a shared normative orientation of the group (Arminen, 2004, p. 691), that is, to not express their ethnic or national identity.

**Figure 3:**
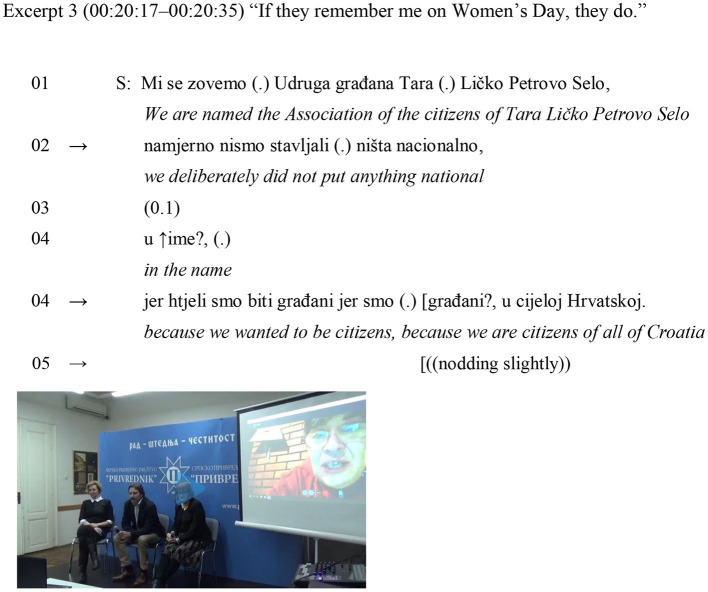
Excerpt 3 – This video features Sonja Leka, a handcraft entrepreneur with the Association of Citizens of Tara (participating via Skype), Rada Borić, a representative of the liberal left *Možemo!* [We Can!], and Dragana Jeckov, a parliamentarian from the Independent Democratic Serb Party (SDSS). A moderator for the event is Saša Kosanović (Privrednik, 2018a).

The discussants are Sonja Leka, a handicraft entrepreneur with the Association of Citizens of Tara (participating through a Skype video call), and the moderator is Saša Kosanović (Privrednik, 2018a; Figure 3).

**Figure 4:**
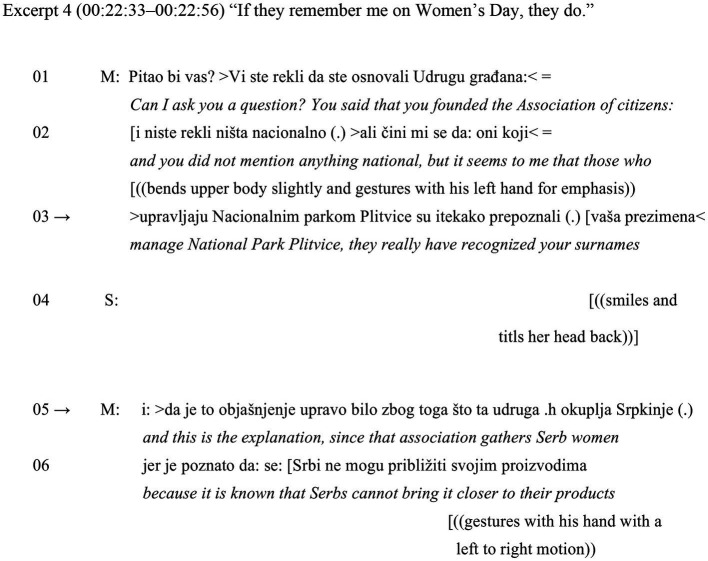
Excerpt 4 – (see Excerpt 3).

The moderator in Excerpt 4 encourages her to evaluate this statement when he asks lines (4.1–4.5), “Can I ask you a question? You said you founded the Association of Citizens, and you did not mention anything national, but it seems to me that those who manage National Park Plitvice, they really have recognized your surnames.” Sonja’s response occurs in line (4.6) as she “smiles and tilts her head back.” In this manner, she appears to propose that disclosing one’s ethnic identity is not a serious matter or that the equality laws have been overapplied as outlined in the Charter of Fundamental Rights (European Union, 2016; Figure 4).

Let us consider Article 52 of the EU Charter of Fundamental Rights: “Any limitation on the exercise of the rights and freedoms recognized by this Charter must be provided for by law and respect the essence of those rights and freedoms” (European Union, 2016). This downgrading of the rule is further supported by a reference to the group’s shared normative orientation in lines (4.7–4.8): “and this is the explanation, since that association gathers Serb women, because it is known that Serbs cannot bring it closer to their [Croat] products.” In addition, the moderator points out that the Association produces woolen socks and hats, and he is compelled to mention that they also offer exceptionally high-quality woolen penis warmers called *nakurnjaci*, which he would recommend to men who like this traditional somewhat forgotten, but extremely useful, item of clothing. As we can see here, the moderator focuses on overt transgression and provides his reasoning for sanctioning it (Arminen, 2016, p. 691). In her response, Sonja does attempt to refute some of the moderator’s statements by remarking that now the situation is different and that people on the national team who sell are mixed; it has improved but with difficulty.

### 3.4 Participants’ ambivalence regarding ethnicity and gender equality surfaced through laughter

The topic of this excerpt, “Ethnic division of young people–so close, yet so far,” is focused on the failed attempt at establishing an intercultural school “Dunav” or Danube in Vukovar. This is a city in Eastern Slavonia that was destroyed during the Croatian War of Independence. Initially, the idea came from the Norwegian Nansen Dialog Center, but it was not well accepted by the local parents’ community and political leaders in Vukovar (Paravina, 2022, p. 9). Dragana Jeckov, a Parliament representative for the Independent Democratic Serb Party [*Samostalna Demokratska Srpska Stranka* – SDSS], introduces the issue of gender and ethnic inequality. In lines (5.14–5.17), she states: “I am interested who would be the aunt who cleans, I am interested who would be a female cook who gives tea bread milk.” The moderator is clearly not aligning with Dragana’s statements as evidenced by his comment in line (5.19): “How? Whose?” which is designed to cause Dragana discomfort because it occurs structurally after Dragana has initiated a turn which is legitimately hers to take (Wooffitt, 2005, p. 174). The moderator’s speech overlaps with the word “milk” line (5.17).

**Figure 5:**
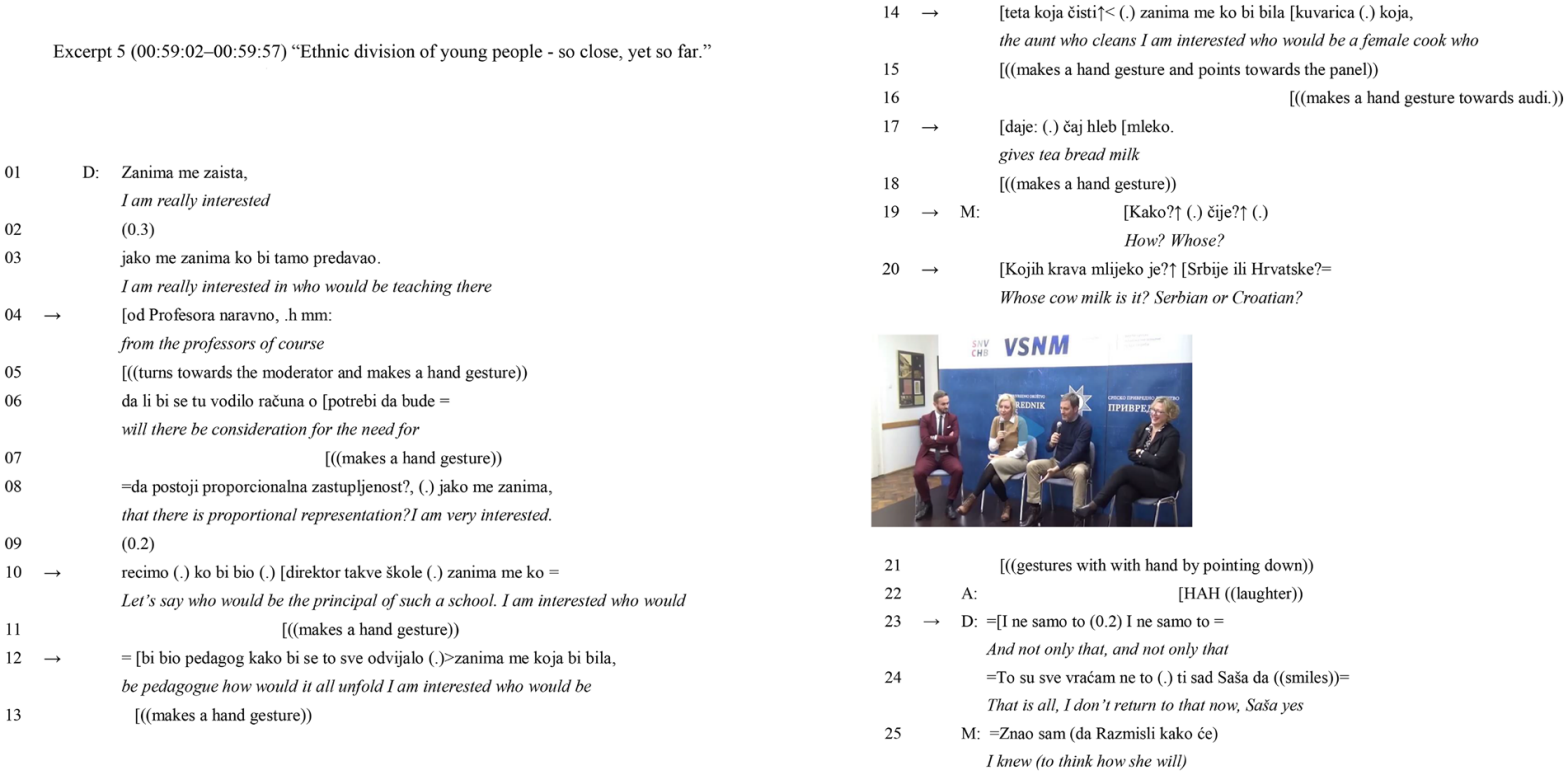
Excerpt 5 –˙ This video features Jovan Vlaović, President of the Committee for Human Rights, Dragana Jeckov, a parliamentarian from the SDSS, Dinka Čorkalo Biruški, Professor at the Faculty of Philosophy in Zagreb, and moderator Saša Kosanović (Privrednik, 2018b).

The discussants are Dragana Jeckov, a parliamentarian for SDSS, and the moderator is Saša Kosanović (Privrednik, 2018b; Figure 5).

Dragana constructs a gendered context out of previously referring to masculine gender words for professions such as “professor,” “principal,” and “pedagogue” lines (5.4, 5.10, 5.12) to female gendered ones lines (5.14–5.17): “aunt who cleans,” and “a female cook who gives tea, bread, milk” (Stokoe, 2012b, p. 239). The moderator then formulates an upshot (line 5.20) “Whose cow milk is it? Serbian or Croatian?” It may have been intended to diminish the care work such as the preparation of food by women. In this manner, the moderator presents a blatant violation of the rule (Arminen, 2004, p. 698) that invites some roaring laughter line (5.22).^8^

If we consider Article 23 to be the social order here, then as it stipulates: “The principle of equality shall not prevent the maintenance or adoption of measures providing for specific advantages in favor of the under-represented sex” (European Union, 2016). As Arminen (2004, p. 697) observes in his study, there are two types of rules of authority. The first are rules that reaffirm everyday moral standards and are left without further clarification. The second type consists of rules that are contingent upon the institutional setting that unfold so that parties can take into account the contingencies involved. Furthermore, participants do not treat these rules equally; mundane normative standards are taken for granted while institutional rules are held accountable (Arminen, 2004, p. 697). Tension arises when these two subsets of rules do not integrate, and this occasionally generates laughter. In this instance, it appears that the moderator, the audience, and Dragana do not identify category knowledge in the same manner, indicating that they do not share the same culture (Stokoe, 2012b, p. 240). Furthermore, in order to present oneself as a member of a social group, both propositional knowledge and the ability to interpret that knowledge are necessary (Arminen and Heino, 2022, p. 224). Dragana does not initiate a repair, but she is further interrupted by the moderator as she attempts to complete her statement line (5.23): “And not only that, and not only that.” As an upshot or punchline line (5.20), the question, “Whose cow milk is it? Serbian or Croatian?” violates the institutional turn-taking rule and invites laughter, but it also illustrates an everyday cultural competence that is used to juxtapose the institutionally established order (Arminen, 2004, p. 698). In short, the discussion on Article 23 on the equality between women and men in all areas (including employment, work, and pay) is being laughed at in an environment that should provide a safe space for this subject to be discussed.

## 4 Discussion

This analysis demonstrates through intersectionality how members of the Serb ethnic minority invoke, produce, and propose their common knowledge of ethnicity and gender. This research documents that ethnic membership in Croatia can be understood as either a plain reading of the ethnicity and gender rights, contextualization or as a personalization of these rights, and finally a downgrading of the minority rights through reformulation (Arminen, 2016). Sociology and anthropology have studied ethnicity and gender (Evans-Pritchard, 1940; Eidheim, 1969; Malkki, 1995) and how people’s sense of living in ethnic groups is contingent on the situational maintenance of boundaries, which can be crossed in multiple ways. The present study discusses the CA corpus and scholarship of Arminen (2004) study and through the orientation by Arminen, has discovered the unfolding and intersection of these aspects of ethnic membership through gender, age, class and professional. By adopting both ethnomethodological approaches MCA and CA to determine the manner in which Serb women orient to ethnicity, gender, and intersectionality as aspects of my study, this analysis has uncovered the stigma as well as the construction of a gender category in a culture that is demeaning (Stokoe, 2012b). In an empirically grounded manner, Excerpts 1 and 5 demonstrated a distinct *categorical practice* of describing women that is not an intangible stereotype, but rather a set of sequences which progress on the basis of presumed shared knowledge (Stokoe, 2012b, pp. 249–250). This description-turned-categorization is delivered with a laugh, and it illustrates recognizable cultural knowledge as description-turned-categorization (Stokoe, 2012b, p. 250).

Goffman (1963, p. 9) defines stigma as the situation for the individual who is disqualified from full social acceptance. In Excerpt 1, Tatjana, who is an ethnic Serb, presents the case for others who are stigmatized and is a living model of fully normal achievement, a hero of adjustment “who are subject to public awards for proving that an individual of this kind can be a good person” (Goffman, 1963, p. 37). We can also notice in this excerpt that by “reading the rules plainly,” Tanja accepts and respects herself and feels no need to conceal her ethnic identity. In Goffman (1963, p. 125) terms, she is “above passing.” The term “coming out of the closet” is also associated with the gay and lesbian liberation movements that began in the 1970s. When comparing ethnicity and homosexuality as minorities in society, there are parallels that can be drawn, such as the difficulties of a congenital nature as well as social acceptance (Weeks, 1990, p. 111). In the case of lesbianism, it can also involve an assertion of femaleness and of identity. It is important to mention that the moderator does compare Tatjana to a gay man rather than a lesbian woman when he states in line (1.12) “Some boys say” in reference to “Come out of the closet.” Tatjana’s response at first clearly demonstrates that “categories short-cut and package common sense knowledge” (Stokoe, 2012a, p. 300) when she aligns with the moderator in line (1.11). Nevertheless, during the subsequent turn, Tatjana asserts a more neutral stance towards the moderator and expands the comparison to all “who are not only different in Croatia but all over the world.” In the 1990s, feminist women were believed to be “Witches from Rio” in Croatia for several reasons, including the fact that national ideology and feminism were perceived as mutually exclusive terms (Pavlović, 2002, pp. 147–150). Furthermore, “women as mothers are reproducers of the nation” while the women of others are “multiplying the number of outsiders” and in civil wars, mass rapes have the same analogy that women’s bodies should be defended as borders and territories (Iveković and Mostov, 2002, pp. 11–12). A comparison of a woman with a gay man in the ethnic context is thus problematic. Although those who identify as belonging to the LGB community in Croatia have the same rights as heterosexual couples according to the Same-sex Life Partnership Act, women and gay man are simply not shared categories.^9^

Excerpt 2 constitutes an attempt to encourage the discussant to make a “good adjustment” to be a stigmatized individual who accepts oneself consciously and cheerfully as a normal person (or member of the majority in this context), while at the same time voluntarily avoiding situations where members of a majority would have difficulty accepting that person in the same manner (Goffman, 1963, p.146). By interrupting Bogdanka’s turn, Maša provokes her to reflect on the here and now. However, Bogdanka maintains her stance and continues to talk about her personal experience of stigmatization during the Croatian War of Independence. The manner in which the discussant perceives time can be compared to that of Ssorin-Chaikov (2017, p. 13). Ssorin-Chaikov uses Bergson’s philosophical critique of relativity theory to suggest that there is no single “real” time but rather a set of multiple “real” times concerning the time after the collapse of the Soviet Union. Similar reconfigurations of public spaces, deindustrialization, and significant changes in the local inhabitants lived experiences have not been sufficiently researched in the Croatian context: “the Yugoslav time is historical, while the (post)Yugoslav space and the many people who inhabit(ed) that time and space still exist” (Bonfiglioli, 2020, p. 5). “That’s a terrible blow to a person’s dignity,” observes Bogdanka, and in this case, we can note that she used the word “*čovjek*” as a “quasi-non-referential,” using the second person instead of the first to place herself in the background and generalize, but also to connect both herself and the interlocutor and involve them in her experience (Kordić, 2001, p. 471).

Bogdanka also describes vividly how forced labor was a horrible reminder of the Jasenovac concentration camp because all of the workers were either Serbs or had Serb partners. Throughout her scholarship, David (2020, p. 1, p. 6) traces “the emergence of the ‘dealing with the past’ agenda” since the 1970s where various groups attempt to “fight impunity and tackle difficult legacies” through transitional justice or historical justice. However, this also entails some negative aspects, such as “competition over victimhood,” resulting in differing access to resources (David, 2020, p. 11). This also “reduces pasts to one singular remembrance frame” such that Srebrenica victims of genocide are regarded as “prototype for victims” internationally and locally (David, 2020, pp. 9–10). At the same time, Bogdanka’s statement on the loss of dignity in labor brings to mind the links between the word “*čovjek*” and a “man”, thus further illustrating how gender roles based on an economic “male breadwinner” model may negatively impact men’s sense of self. Pfau-Effinger (2004, p. 394) mentions the socio-historical paths of the male breadwinner model in Germany, the Netherlands, and Finland shows the significant role of the urban bourgeoisie in the development of the male breadwinner model. Finland is an exception because it adopted the ideal of a family economy model with a relatively egalitarian structure with women integrated into occupational employment combined with childcare provided by the welfare state (Pfau-Effinger, 2004, pp. 392–393). Gender relations are highly impacted by the national welfare regimes and state policies, and although East European ex-socialist countries were characterized by the universal state provision, since the late 1980s, they have been making the often uncomfortable provision to market capitalism (Crompton et al., 2007, p. 7). Furthermore, Crompton et al. (2007, p. 6) maintain that gender relations are also impacted by the domestic division of labor/class, level of education, and ethnic differences. It is therefore relevant to contextualize and personalize ethnic identity in this regard.

Goffman (1963, pp. 125–126) argues that even if people are willing to admit they possess stigma (often because it is known about or obvious), they may nonetheless attempt to prevent that stigma from having greater significance. In this manner, the individual aims to reduce tension and maintain spontaneous involvement in the formal content of the interaction. However, the means used for this are similar to “passing.” As we can see in Excerpt 3, Sonja does not present the association that produces knitted work and its members as Serb women but as Croatian citizens. The moderator does remind her that the members’ surnames make their identities rather obvious. Goffman (1963, p. 125) describes voluntary disclosure as belonging to the mature, well-adjusted state of grace, which is the final stage of the moral career. An interesting aspect of this identity disclosure is that one of the products that is made by the association is sexually suggestive and somewhat transgressive within the traditional, perhaps conservative context of the Plitvice Lakes. Associated with this may be a sense of downgrading the rules and what Goffman (1963, p. 160) describes as the use of self-abusive language and style by representatives of the groups. Another aspect of this is deviance. In this respect, Goffman (1963, p.167) maintains the following: “Social deviants, as defined, flaunt their refusal to accept their place and are temporarily tolerated in this gestural rebellion, providing it is restricted within the ecological boundaries of their community.”

During the last quarter century, Neoliberalism has been a dominant economic and cultural system that has placed “personal responsibility” as the central moral principle, and it requires people to ignore the needs of others (Tronto, 2017, pp. 29–30). Occupational, social, and political factors have led to a rejection of vulnerability, which decreases emphatic capacities and personal responsibility (Tronto, 2017, p. 34). For this reason, we must view care in its much broader context. We must envision care not only as involving economic actors but as Tronto (2017, p. 37) framed *homines curans*, those who conceive of democratic caring as an alternative to neoliberalism, which requires that resources be more equally shared so as to ensure that burdens and benefits are distributed. In that sense, citizenship cannot only be claimed through engaging in the neoliberal market economy, and the perception that the individual worker remains somehow autonomous and that his or her work ethic is not dependent on equality of opportunity but on how hard one works (Tronto, 2013, p. 86). Sonja’s statement about being citizens of all of Croatia reflects that view. In feminist ethics of care, one of the important aspects is the understanding that care not only concerns professional care, institutional care, or at-home care, but also involves a societal shift in understanding the value of care, that care relations are interdependent; they are personal, political and central to sustaining societies as well as human and planetary life (O’Riordan et al., 2023, p. 6).

Excerpt 5 also highlights the importance of the intersectional approach in relation to ethnicity and gender. The Council of Europe Gender Equality Strategy 2018–2023 emphasizes that “Intersectional discrimination on the grounds of ethnicity, age, disability, sexual orientation or gender identity, among others, disproportionately marginalizes particular groups of women” (Council of Europe, 2018, p. 11). Several studies have reported that prejudices and stereotypes in the division of labor at home affect women’s academic careers and achievement in Serbian higher education, regarding institutional mechanisms and policies of gender inequality (Babović et al., 2019, p. 164). Psychologists are overwhelmingly male professors in Serbian universities, and certain academic fields are still gender-segregated (Babović et al., 2019, p. 164). In addition, gender equality studies have reported that Croatia scored “slightly worse in the knowledge and power” domains; “knowledge here refers to attainment and participation (tertiary and formal/non-formal education) and segregation (tertiary students, health and welfare, humanities and arts)” (Miković, 2020, p. 66). Gender equality must concern both men and women, as the focus has been primarily on “women as the problem,” but it must include “the problem of men,” and “further gender/sexual categories, for example, LGBTIQ+, including non-binary, agender and asexual categories” (Hearn, 2019, p. 28). Some of the behavior that men can adopt are “not interrupting the person who is speaking,” becoming a good listener,” “not speaking on every subject,” and “not putting others down” (Hearn, 2019, p. 38). Finally, men should recognize the importance of communication and accountable actions.

## 5 Conclusion

Through the study of institutional interaction in Privrednik, my main objective was to demonstrate how Serb participants interpret, discuss, and achieve what they consider to be correct. As elaborated on in Arminen (2016, p.75), I have outlined a sequential CA pattern that is similar to Arminen (2004) study of institutional rules. This includes (1) the reading of ethnicity and gender rights plainly; (2) contextualizing ethnicity in a personalized manner; (3) the downgrading of ethnicity rights through reformulation; and (4) laughter reflecting ambivalence towards gender equality and ethnicity. The limitation of this study is that a larger dataset may have provided a better indication of the distribution of patterns. Furthermore, I have also revealed how ethnicity and gender intersect to shape the social worlds of participants in the Privrednik’s public discussion forum. This aspect of my analysis also demonstrates the stigma (Goffman, 1963) and categorical practices in a description of women based on presumed shared knowledge as outlined in the MCA study by Stokoe (2012b). I venture to suggest that the continuum of separatism and inclusionary organizing can benefit Privrednik in order to navigate and limit the effects of ethnic, gendered, and sexualized power relations in post-ethnic activism (Keskinen, 2022, p. 65). Apart from providing a relatively comfortable and safe environment for discussion, the activities allow groups to concentrate on the people for whom they are intended.

## Data availability statement

The original contributions presented in the study are included in the article, further inquiries can be directed to the corresponding author.

## Ethics statement

Ethical approval was not required for the study involving human participants in accordance with the local legislation and institutional requirements. Written informed consent was obtained from the individual(s) for the publication of any potentially identifiable images or data included in this article.

## Author contributions

The author confirms being the sole contributor of this work and has approved it for publication.

## Footnotes

^1^This paragraph and parts of the introduction section are slightly revised from my 2023 conference paper titled “A Discussion of Gender, Ethnicity, and Intersectionality at the Serb Business Association Forum” *CLARC 2023.* Center for Language Research, University of Rijeka.

^2^Terms such as post-socialism and the postwar period evoke associations with the transition which is, according to economic terms, perhaps complete as stated by the World Bank and International Monetary Fund; however, the transition period is called upon to explain certain social conditions and in some respects, it is an ephemeral process that continues with new forms of transition that are related to the green environment or health (Kupsjak, 2022).

^3^*Privrednikova tribina: Reci što misliš!* https://privrednik.hr/tribina/ (Accessed November 27, 2023).

^4^The genocide of Serbs in WWII is a highly complex topic beyond the scope of this article. After reviewing the historical and legal literature, I will summarize some of the main points of contention related to the current academic discussions and movement to outlaw the crime resulting in the Convention, which was adopted in 1948 at the General Assembly of the United Nations (1948). The Convention on the Prevention and Punishment of the Crime of Genocide [Online]. Available: https://www.un.org/en/genocideprevention/genocide-convention.shtml [Accessed September 17, 2023]. See also Irvin-Erickson (2016). Croatia currently observes January 27 as Holocaust Day, August 2 as the International Day of Remembrance of Roma Holocaust Victims and April 27 is commemorated by Jasenovac as the day of “Remembrance of all the victims of the Holocaust, genocide, and other victims of fascism” during World War II (David 2017).

^5^Sonix. https://sonix.ai/ (Accessed November 27, 2023).

^6^More detailed information regarding the use of Serbian and Croatian among the Serb minority in Croatia can be found in Paravina (2022).

^7^The film “*Srbenka*” deals with the subject of peer violence against children of different nationalities in Croatia, which in this case pertains to Serbs. The title of the movie is a somewhat pejorative term for a female Serb. The grammatically correct form would be *Srpkinja* but because Croatian and Serbian both use feminine suffixes such as -*ka*, −*kinja*, −*ica*, it appears to also be a pun on the proliferation of neologisms in Croatian and the feminization of language, and how it is occasionally difficult to know which suffix should be used. The study has demonstrated that -*ka* is the most commonly used suffix for forming feminine neologisms in the Serbian language; *−inja* is almost never used, while in Croatian and Slovenian, it is highly productive (Dragićević 2022, see also Filipović and Kuzmanović Jovanović, 2019).

^8^By rule, I refer to Article 23 on the “Equality between women and men” European Union (2016).

^9^See *Zakon o životnom partnerstvu osoba istog spola*. July 28 2014 (last amended October 16 2019). Available at: https://narodne-novine.nn.hr [Accessed November 13 2023]. In accordance with amendments to the Foster Care Act (*Zakon o Udomiteljstvu*) adopted by the Constitutional Court on January 29 2020, same-sex partners can foster, but not adopt. In its statement, the court points out that courts and other authorities must interpret and apply the disputed legal provisions not mentioning same-sex partners to ensure that foster care can be accessed by all persons under equal conditions (see pp. 40–41).
