# Supplementary material for: A discussion of gender, ethnicity, and intersectionality, at the Serb Business Association forum
Source: Front Sociol. 2024 Jan 4;8:1231050. doi: 10.3389/fsoc.2023.1231050 (PMC10794643; doi:10.3389/fsoc.2023.1231050)
Supplement: Supplementary file 1 [file Data_Sheet_1.pdf]

Supplementary Material

A discussion of gender, ethnicity, and intersectionality, at the Serb Business Association forum

Aleksandra Paravina*

*** Correspondence:** Corresponding Author: aleksandra.paravina@helsinki.fi

1 Supplementary Data

Annex 1:

Transcription Symbols

[ ] simultaneous speech and voices, its start and end

= immediately continuous talk, no interval

(0.6) pause and its length in seconds

(.) micropause, shorter than 0.2 seconds

.h in-breath

hh out-breath

_ emphasis

: stretch

YES loud

. falling intonation

, continuing intonation

? rising inflection, not necessarily a question

?, weak rise in intonation

↑ marked rise in pitch

↓ marked fall in pitch

da- production of word is cut off

word< abruptly finished, but not cut off

> < pronounced faster than the surrounding speech

< > pronounced slower than the surrounding speech

$ laughter in the voice

@ @ animated voice

° ° diminishing voice

shivering voice

hah laughter

(word) unclearly heard

(( )) researcher's comment

→ target line; crucial instance for the analyzed speech

The transcription symbols are taken from Arminen (2016, pp. 257–258).

2 References:

Arminen, I. (2016). *Institutional interaction: Studies of talk at work.* New York: Routledge.
